# Systems-level differential gene expression analysis reveals new genetic variants of oral cancer

**DOI:** 10.1038/s41598-020-71346-7

**Published:** 2020-09-04

**Authors:** Syeda Zahra Abbas, Muhammad Imran Qadir, Syed Aun Muhammad

**Affiliations:** Institute of Molecular Biology and Biotechnology, Bahauddin Zakariya University, Multan, Pakistan

**Keywords:** Oral cancer, Computational biology and bioinformatics

## Abstract

Oral cancer (OC) ranked as eleventh malignancy worldwide, with the increasing incidence among young patients. Limited understanding of complications in cancer progression, its development system, and their interactions are major restrictions towards the progress of optimal and effective treatment strategies. The system-level approach has been designed to explore genetic complexity of the disease and to identify novel oral cancer related genes to detect genomic alterations at molecular level, through cDNA differential analysis. We analyzed 21 oral cancer-related cDNA datasets and listed 30 differentially expressed genes (DEGs). Among 30, we found 6 significant DEGs including CYP1A1, CYP1B1, ADCY2, C7, SERPINB5, and ANAPC13 and studied their functional role in OC. Our genomic and interactive analysis showed significant enrichment of xenobiotics metabolism, p53 signaling pathway and microRNA pathways, towards OC progression and development. We used human proteomic data for post-translational modifications to interpret disease mutations and inter-individual genetic variations. The mutational analysis revealed the sequence predicted disordered region of 14%, 12.5%, 10.5% for ADCY2, CYP1B1, and C7 respectively. The MiRNA target prediction showed functional molecular annotation including specific miRNA-targets hsa-miR-4282, hsa-miR-2052, hsa-miR-216a-3p, for CYP1B1, C7, and ADCY2 respectively associated with oral cancer. We constructed the system level network and found important gene signatures. The drug-gene interaction of OC source genes with seven FDA approved OC drugs help to design or identify new drug target or establishing novel biomedical linkages regarding disease pathophysiology. This investigation demonstrates the importance of system genetics for identifying 6 OC genes (CYP1A1, CYP1B1, ADCY2, C7, SERPINB5, and ANAPC13) as potential drugs targets. Our integrative network-based system-level approach would help to find the genetic variants of OC that can accelerate drug discovery outcomes to develop a better understanding regarding treatment strategies for many cancer types.

## Introduction

Oral Cancer constitutes approximately 90% among all Head and Neck Cancer (HNC) sub-types^1^. However, it is more prominent in urban areas of South Asia with a ratio of 15–40% among all cancer types^2^. In Pakistan, it ranked as 2nd most prevalent cancer-type, with increasing incidence in the past few years^3,4^. The complexity of genetic mechanisms in cancer has been revealed through recent investigations. Many biological systems seem to involved in the development and progression of the cancer. But, the complications in system-interactions are limitedly understood which is a major restriction in developing effective treatments^5^. The gene expression studies may help to investigate the differential expression of genes in different biological states, cell cycle stages, subjects or tissues. This gene expression analysis is an important pinpoint for investigating biological processes and their functional disorders. cDNA microarrays were used to monitor and reveal the expression level for thousands of the genes which are differentially expressed in tumors simultaneously^5^. This technique can exploit this valuable information regarding gene expression analysis. This rapidly progressing technique provides comprehensive data for gene expression profiling of thousands of genes to the investigators in one experiment. Many studies demonstrated that this technique is useful to identify novel genes for cancer and its molecular level classification in human^6,7^. Thus, this novel technique may help us to identify new potential targets for drug development for optimal and effective disease therapies. It may also establish an important link between clinical medicine and gene sequences for humans^8^. Gene Expression Omnibus (GEO) is a publicly available database that can be used for high-throughput screening of molecular variants. It contains the microarray data including single and multiple channel-based experiments to determine abundance of genomic DNA, mRNA and protein molecules. The scientific community widely uses the gene expression database to analyze and search the molecular components for systems-level investigations. Several microarrays applications for meta-analysis are designed to investigate pathological mechanisms associated with genetic risk factors^9–11^. Therefore, the genome expression analysis may help to identify unusual alterations across the genome using microarray technology^12^. This study aims to identify genetic causes and some probable genetic variants of oral cancer that will help to modify the therapeutic strategies.

## Results

The cDNA datasets used in this study belong to various normal or cancerous oral tissues and cell lines. The list of datasets used in this study are shown in Table 1.

**Table 1:** List of cDNA datasets analyzed in this study.

S. no	Geo ID	Sample count (case: control)	Platform used	Tissues
1	GSE2280	22:05	GPL96[HG-U133A] Affymetrix Human Genome U133A Array	Oral lymph node
2	GSE3524	16:04	GPL96[HG-U133A] Affymetrix Human Genome U133A Array	Oral squamous
3	GSE10063	4:30	GPL570[HG-U133_Plus_2] Affymetrix Human Genome U133	Keratinocyte cell
4	GSE13601	31:27	GPL8300 [HG_U95Av2] Affymetrix Human Genome U95 Array	Tongue
5	GSE21866	2:03	GPL201 [HG-Focus] Affymetrix Human HG-Focus Target Array	Tongue squamous
6	GSE32142	2:02	GPL570 [HG-U133_Plus_2] Affymetrix Human Genome U133	Oral squamous
7	GSE36111	5:00	GPL571 [HG-U133A_2] Affymetrix Human Genome U133A 2.0 Array	Oral squamous
8	GSE38058	4:00	GPL570 [HG-U133_Plus_2] Affymetrix Human Genome U133	Oral squamous
9	GSE38517	9:11	GPL570 [HG-U133_Plus_2] Affymetrix Human Genome U133	Fibroblasts
10	GSE39376	11:17	GPL201 [HG-Focus] Affymetrix Human HG-Focus Target Array	Buccal carcinoma cell
11	GSE43862	1:03	GPL570 [HG-U133_Plus_2] Affymetrix Human Genome U133	Oral squamous
12	GSE44458	4:00	GPL570 [HG-U133_Plus_2] Affymetrix Human Genome U133	Tongue squamous
13	GSE49673	6:06	GPL570 [HG-U133_Plus_2] Affymetrix Human Genome U133	Parotid adenocarcinoma
14	GSE52811	4:04	GPL8786 [miRNA-1] Affymetrix Multispecies miRNA-1 Array	Oral keratinocyte
15	GSE52915	27:00	GPL570 [HG-U133_Plus_2] Affymetrix Human Genome U133	Tongue squamous
16	GSE57022	2:02	GPL570 [HG-U133_Plus_2] Affymetrix Human Genome U133	Parotid adenocarcinoma
17	GSE59795	10:10	GPL570 [HG-U133_Plus_2] Affymetrix Human Genome U133	Parotid adenocarcinoma
18	GSE70301	3:03	GPL570 [HG-U133_Plus_2] Affymetrix Human Genome U133	Tongue squamous
19	GSE73171	3:03	GPL14613 [miRNA-2] Affymetrix Multispecies miRNA-2 Array	Laryngeal squamous
20	GSE75127	4:4	GPL570 [HG-U133_Plus_2] Affymetrix Human Genome U133 Plus 2.0 Array	Oral squamous
21	GSE81821	5:5	GPL14613 [miRNA-2] Affymetrix Multispecies miRNA-2 Array	Metastatic tumor tissue

### Differential analysis, normalization and cross-validation

**Figure 1:**
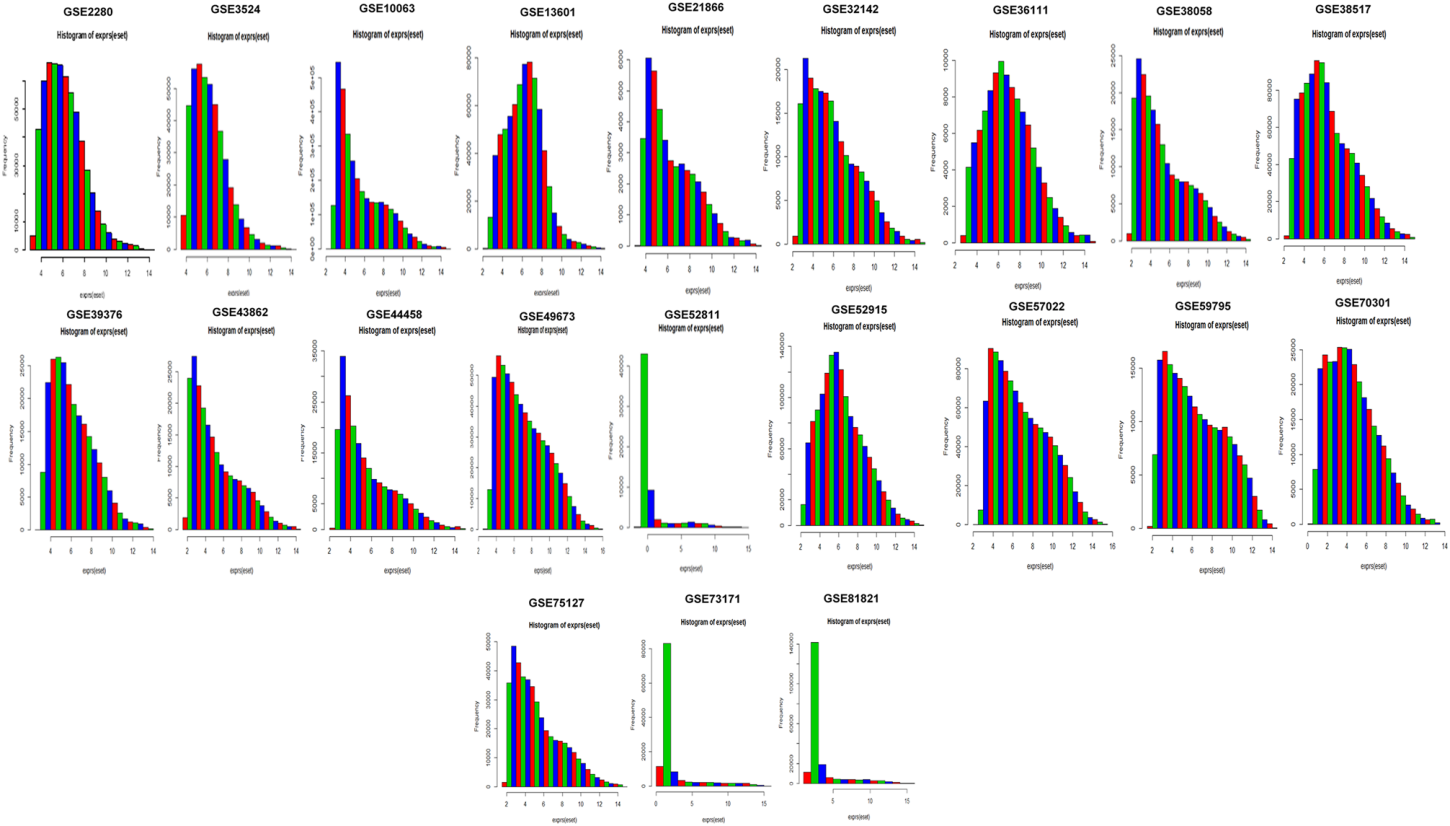
Normalization and differential analysis. Histogram (smoothed histograms) shows density estimate of the data. Typically, the distributions of the arrays have similar shapes and ranges. Arrays whose distributions are very different from the others are considered for possible problems. High levels of background shifted an array's distribution to the right. Lack of signal diminishes its right tail. A bulge at the upper end of the intensity range indicated the signal saturation.

**Figure 2:**
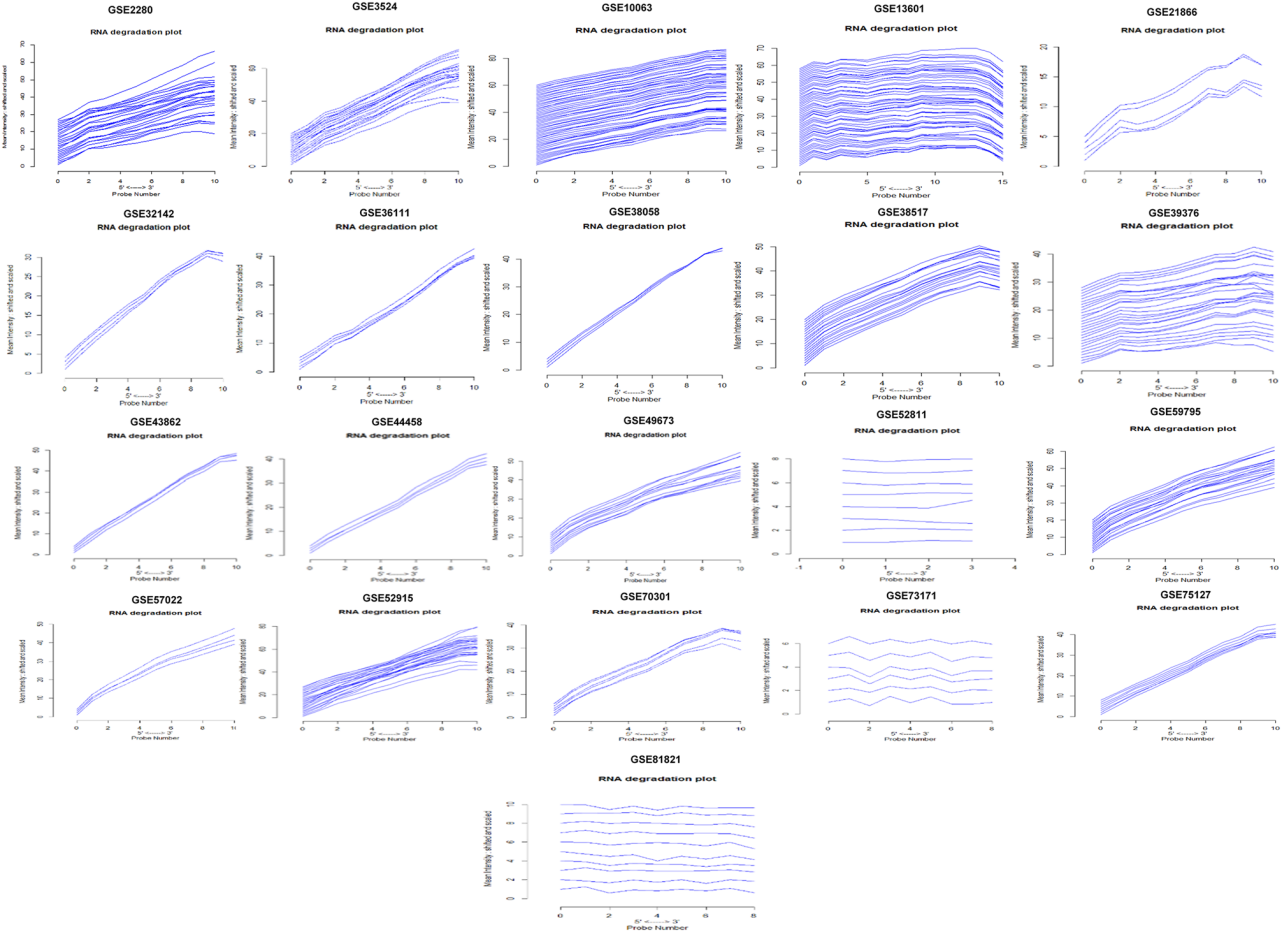
RNA degradation plot. Side-by-side plot produced by plot AffyRNAdeg representing 5′–3′-trend indicating an assessment of the severity of RNA degradation and significance level.

We analyzed 21 cDNA datasets specifically associated with oral cancer. Each dataset has a different number of samples and the genes derived through mRNA expression profiling using different Affymetrix platforms for OC. The histograms representing expression after normalization indicate the density estimation of data. The shapes and ranges for arrays distributions are similar indicating the quality of the data. The array’s distribution towards the right shows a high background level. The saturation of signal is specified by bulge which appears at the upper end of intensity range (Fig. 1). An automated mechanism was used to perform a comparison of biologically similar groups in pairs. We let off any subgroup without repetition from comparisons for accuracy and verification of differential analysis, and the generalized linear models' 'cv.glm' method measured the error of the cross-validation prediction. The Gaussian dispersion criterion is 0.00519 which indicates the degree of confidence (Table 2). With K-folds estimation we obtained the same delta value of 0.00515 as we used the LOOCV approach (during raw cross-validation and afterward during modified cross-validation). The substantial codes (0.1, 0.01, 0.001, and 0.05) with residuals of limited deviance suggested the consistency of the differential analysis. cDNA datasets were also analyzed for some necessary factors like RNA quality, sequence biases or RNA degradation. In genomic analysis, the use of low-quality RNA samples in the sequencing of the entire genome is inefficient. It is not clear if transcript degradation occurs reliably in low-quality RNA samples, in which case the effects of degradation can be reversed by data normalization or whether different RNA samples can be degraded at different rates, which could bias expression measurements. So, for differential expression analysis, we assessed the RNA quality. To verify the dataset reliability for identification of variation at the transcriptional level in original samples. The normalization process was used to standardize sample handling techniques and to assess optimal RNA variability threshold by using discrimination measures for statistical and algorithmic analysis. All the probe sets have their individual probes aligned at 5′-end of the target RNA molecule. The competitive binding of a particular probe to its target has been observed to depend upon a 3′/5′ intensity gradient. Due to the poor quality of RNA, a reduced quantity of RNA is hybridized to the array. The low hybridization leads to a decrease in the total signal output level. But if the degree of saturation level increases the 3′/5′ intensity gradient decreases. The 3′-end of the target gene contains a probe set that corresponds to the transcripts. The statistical and function summary for each batch-array is produced by ‘AffyRNAdeg’ to measure RNA degradation level and its significance (Fig. 2).

**Table 2:** K-fold Cross-validation using “Boot” package of bioconductor software based on Gaussian dispersion modules.

	**Estimate**	**Std. error**	**t. value**	**Pr( >|t|)**
(Intercept)	0.021148	0.003402	6.17	< 1.00E^−14^***
x1	0.052155	0.003825	22.009	< 1.00E^−14^***
x2	− 0.02651	0.005117	− 7.027	< 1.96E^−09^***
x3	0.149112	0.003105	30.007	< 1.00E^−14^***
x4	0.216532	0.001828	21.510	< 1.00E^−14^***
x5	0.048403	0.002152	31.003	< 1.00E^−14^***
x6	0.132542	0.002071	25.001	< 1.00E^−14^***
x7	− 0.07733	0.001672	− 29.216	< 1.00E^−14^***
x8	0.121504	0.002002	20.124	< 1.00E^−14^***
x9	0.212811	0.002825	65.278	< 1.00E^−14^***
x10	0.010029	0.003708	6.335	< 1.00E^−14^***
x11	0.022541	0.007523	22.601	< 1.00E^−14^***
x12	− 0.02215	0.001205	− 5.011	0.0089*
x13	− 0.12532	0.003051	− 56.055	< 1.00E^−14^***
x14	0.030526	0.001052	3.415	5.28E^−12^***
x15	− 0.028691	0.001611	− 20.502	< 1.00E^−14^***

Deviance residuals: Min (− 1.5101), 1Q (− 0.0412), Median (− 0.0100), 3Q (0.0132), Max (3.1977).

Signif. codes: 0 ‘***’ 0.001 ‘**’ 0.01 ‘*’ 0.05 ‘.’ 0.1 ‘ ’ 1.

Number of Fisher Scoring iterations: 2; $K: [1] 10; $delta: [1] 0.00516 = 0.00515.

Null deviance: 100,813.5 on 53,225 degrees of freedom.

Residual deviance: 2,817.3 on 53,209 degrees of freedom.

### Disease-gene curation for differentially expressed genes (DEGs)

**Figure 3:**
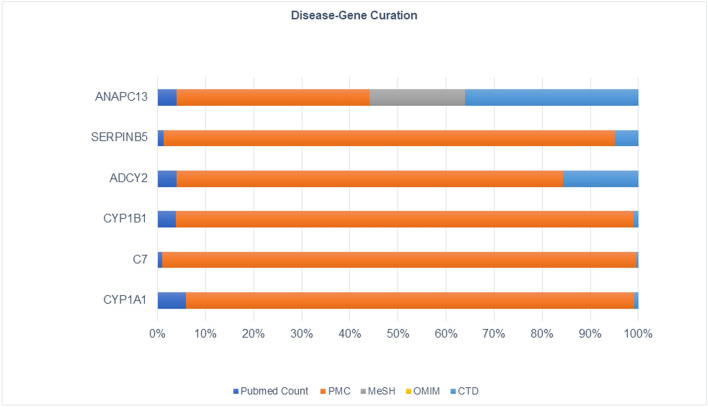
The OC-related DEGs were curated using CTD (comparative toxicogenomics database), PubMed, OMIM, and MeSH databases.

From 21 datasets, we found 30 DEGs and David tool was used to retrieve their gene symbol and biological annotation. We selected the most significant ranked genes from the list of differentially expressed genes. For disease-gene curation, these genes were text mined using CTD (Comparative Toxicogenomics Database), PubMed, OMIM, MeSH, and PMC databases to filter disease-specific genes (Supplementary Table 1). We observed *CYP1B1*, *CYP1A1*, *C7*, *ADCY2*, *SERPINB5*, and *ANAPC13* are the most curated terms in the databases. These shortlisted genes were further analyzed by mapping at (*p* < 0.00005) through Cancer Genetics and OMIM databases and observed their role in carcinogenesis (Fig. 3).

### Enrichment and cluster analysis of DEGs

**Figure 4:**
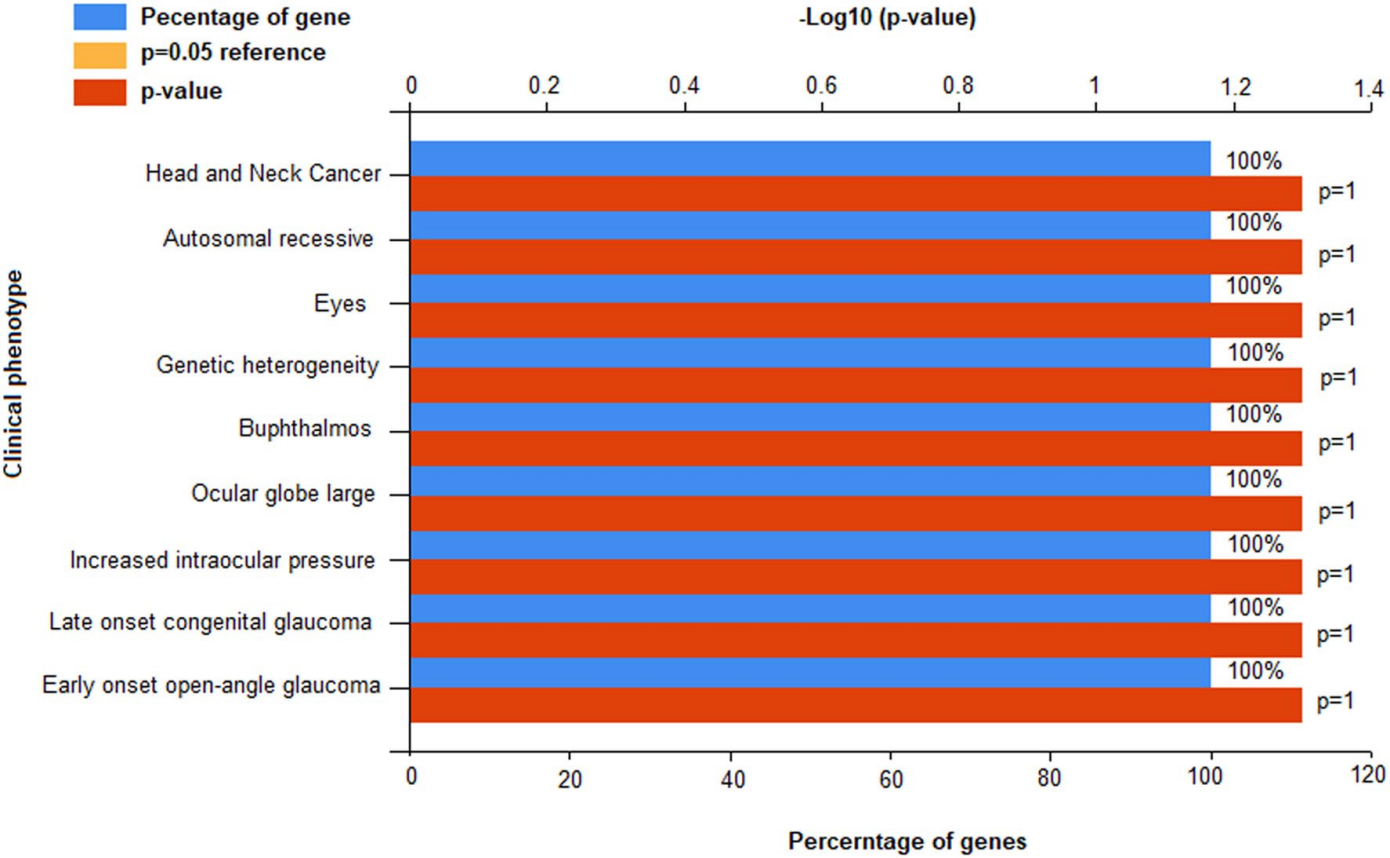
Clinical phenotypes for oral cancer related DEGs using FunRich annotation tool.

These genes showed enrichment substantially linked with hydroxylase, P450 pathway, steroid metabolic process, monooxygenase activity, cellular response to organic cyclic compound, and aromatase activity (Table 3). The dysregulation of these genes causes genetic heterogeneity, autosomal recessive disorder, head and neck disorders and other clinical phenotypes (Fig. 4).

**Table 3:** Gene Ontology and functional enrichment of differentially expressed genes.

Term	*p* value	Fold enrichment	FDR
hsa04913: Ovarian steroidogenesis	4.86E−04	70.5102	0.464991
GO:0,097,267 ~ omega-hydroxylase P450 pathway	2.68E−03	621.9259	2.926305
GO:0,016,712 ~ oxidoreductase activity	0.00355	450.16	2.812419
GO:0,019,373 ~ epoxygenase P450 pathway	0.005349	310.963	5.768479
GO:0,070,330 ~ aromatase activity	0.006383	250.0889	5.007033
GO:0,019,825 ~ oxygen binding	0.011091	143.6681	8.558457
GO:0,008,202 ~ steroid metabolic process	0.01274	130.1705	13.24158
IPR002401: Cytochrome P450, E-class, group I	0.0134	123.7267	9.757773
GO:0,004,497 ~ monooxygenase activity	0.013674	116.4207	10.45655
IPR017972: Cytochrome P450, conserved site	0.01553	106.6609	11.23041
IPR001128: Cytochrome P450	0.017126	96.66146	12.31951
GO:0,071,407 ~ cellular response to organic cyclic compound	0.017447	94.87006	17.71563
Monooxygenase	0.021199	77.95833	17.98805
GO:0,031,090 ~ organelle membrane	0.023645	69.82375	15.90821
Metal ion-binding site: Iron (heme axial ligand)	0.024432	67.55219	18.69242
hsa00380: Tryptophan metabolism	0.028619	57.58333	24.30038
Microsome	0.030952	53.18088	25.24757
Heme	0.031663	51.97222	25.75335
GO:0,020,037 ~ heme binding	0.032072	49.28759	23.01009
Secondary metabolites biosynthesis, transport, and catabolism	0.032577	30.69697	3.257651
GO:0,005,506 ~ iron ion binding	0.035767	44.13333	25.3362
hsa00140: Steroid hormone biosynthesis	0.041281	39.71264	33.24928
hsa00980: Metabolism of xenobiotics by cytochrome P450	0.052426	31.12613	40.32831
hsa05204: Chemical carcinogenesis	0.056578	28.79167	42.78879
hsa04914: Progesterone-mediated oocyte maturation	0.061404	26.4751	45.53397
Glycoprotein	0.075408	3.014869	51.59749
Iron	0.076281	21.17387	52.01866
hsa04114: Oocyte meiosis	0.076443	21.1315	53.34849
hsa04114: Oocyte meiosis	0.076443	21.1315	53.34849

The function of the gene, its regulation, subtypes, and cellular processes play a key role in understanding its biology. The functional enrichment analysis showed that shortlisted CYP1A1, CYP1B1 genes are known to involved in xenobiotic metabolic and energy pathways. C7 is involved in immune response whereas SERPINB5 is known for protein metabolism. While ADCY2 plays an important role in cell communication and signal transduction. ANAPC13, have a potential role in class-I MHC-mediated antigens and cell-cycle progression at early tumor stages. The membrane attack complex in the extracellular region for C7, Expression of CYP1A1, SERPINB5, CYP1B1 is found in the endoplasmic reticulum. CYP1A1 is also found in nucleus and microsomes. ANAPC13 is a well-known anaphase-promoting complex. Differential expression of ADCY2, SERPINB5, CYP1B1 is also found in the cytoplasm.

**Figure 5:**
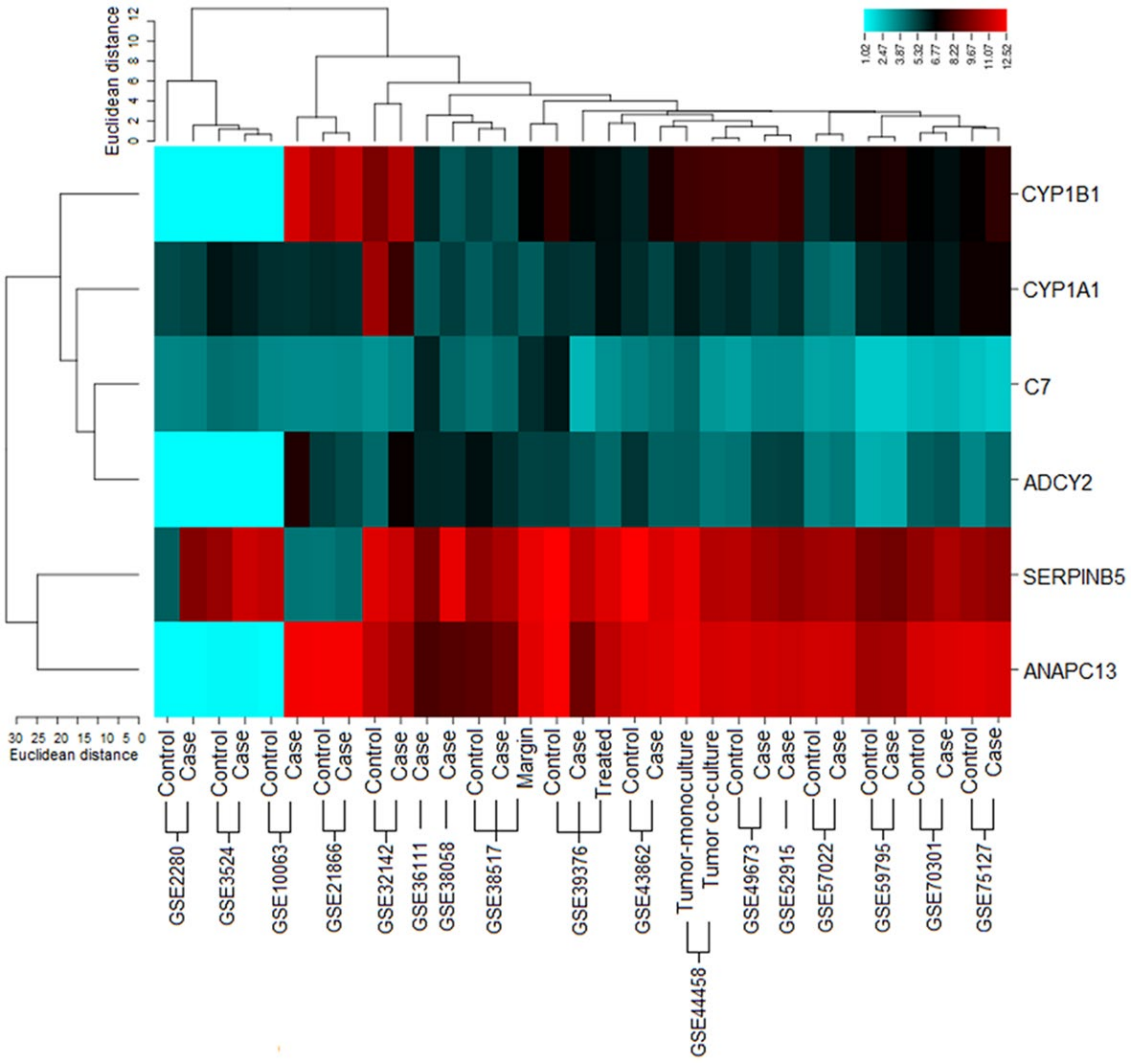
Cluster analysis of 6 oral cancer-related DEGs with Euclidean distance (Binning method). Quantile lines indicate the boundaries of the clusters in the level of the tree.

Cluster analysis of selected DEGs helps us in the recognition of functional annotation and significance. The results were observed with the Euclidean distance (Fig. 5). The genetic expression of sample cells is distinguished as cases and control indicating the obvious differences between two of these groups. The analysis showed the down and up-regulated genes based on the *p* value and fold changes (Table 4).

**Table 4:** Expression profiling of cDNA microarray datasets.

AFFYMETRIX_ 3PRIME_IVT_ID	Gene name	logFC	AveExpr	t	*p* value	adj. *p-*Val	B	Abberation
205749_at	*CYP1A1*	3.900696	8.398162	39.22931	2.99E−46	1.63E−41	87.06766	Up regulated
202992_at	*C7*	− 1.84918	4.492108	− 6.64914	3.77E−07	0.008403	4.18572	Down regulated
202437_s_at	*CYP1B1*	3.140615	8.48289	25.87402	1.89E−35	5.18E−31	67.29499	Up regulated
213217_at	*ADCY2*	− 2.07208	5.693218	− 7.54109	3.51E−07	0.019192	4.699974	Down regulated
204855_at	*SERPINB5*	− 6.46131	9.39441	− 41.2474	2.06E−26	9.05E−23	49.19926	Down regulated
209001_s_at	ANAPC13	− 2.49883	10.52767	− 31.6716	1.32E−10	7.23E−06	13.02867	Down regulated

### Mutation analysis

**Figure 6:**
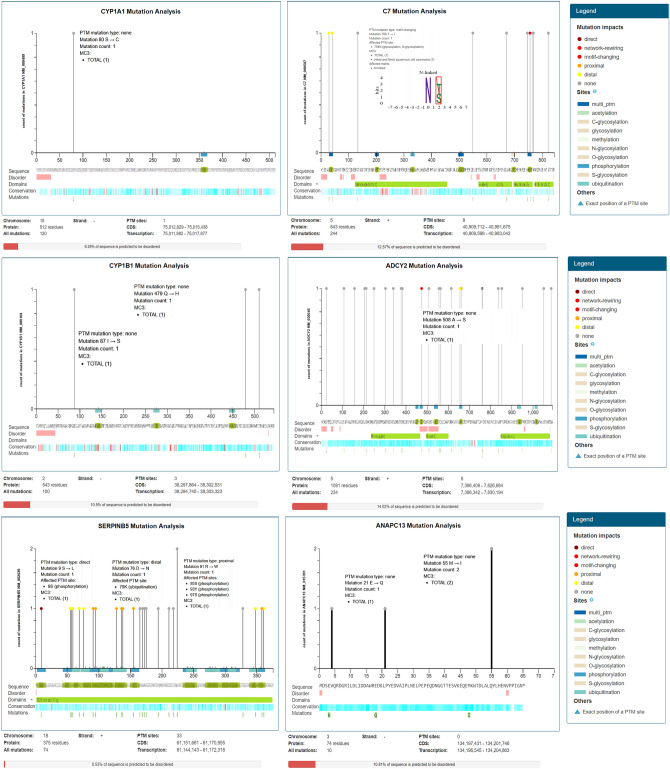
Analysis and exploration of mutations affecting post-translational modification (PTM) sites in human genes/proteins using online ActiveDriverDB database. Needle plots indicate the PTM site mutations in our genes/proteins. The graph shows the outcomes based on the specific type of PTM, cancer type, and mutation subset (presented in legend color codes). Height (y-axis) represents the number of occurrences of the mutation while Horizontal position (x-axis) indicates the position of protein’s amino acid sequence. Pinhead color signifies the mutation impact and X-axis coloring shows the type of PTM associated with the mutation location. Mutational Impacts: Rewiring: mutation-induced gains and losses of kinase-bound sequence motifs (predicted by MIMP software); Distal: mutation affects an amino acid located 4–7 amino acids away from the PTM site; Direct: mutation affects the post transcriptionally modified amino acid; Proximal: mutation affects an amino acid located 1–3 amino acids away from PTM site; Sites: Amino acid sites/ regions enriched for mutations affecting post-translational modifications (PTMs).

ADCY2 has eight post-translational modification (PTM) sites with 234 recurrent cancer mutations at the chromosome no. 5 positive-strand encoding 1091 protein residues representing 14.02% of the predicted disordered region. The mutation visualization plot shows ADCY2 isoform ADCY2 Q474R direct network-rewiring mutation impact at the position 474, with reference amino acid residue Q and mutated amino acid residue R in the protein. The affected-site at position 472 with S amino acid residue-site enriched with a phosphorylation-type mutation affecting PTMs. Another, ADCY2 isoform ADCY2 S655R, reveals the mutation for this protein at position 655 including amino acid residue S comparison with the mutated amino acid residue R. In position 659, S-amino acid residue site enriched with phosphorylation-type mutation, this shows distal-mutation PTM impact with the affected-site. CYP1B1 showed a 10.5% predicted disordered sequence region with 100 mutations observed at chromosome no 2 on the negative strand with 543 protein residues and 3 PTM sites. CYP1B1 I87S, CYP1B1 Q479H, CYP1B1 T510I isoforms were revealed for CYP1B1 mutational enrichment, at the positions 87,479,510 respectively. The reference amino acid residue for these isoforms was I, Q, T along with mutant amino acid residues S, H, I respectively for each isoform. Similarly, the mutational analysis of C7 showed that 12.57% of the sequence predicted for disease-pathophysiology. Total 244 number of mutations were found on the positive strand of chromosome no. 5 for C7. The number of PTM sites for C7 was eight with 843 protein residues. So, 10 isoforms for C7 were found, among them C7 Q29R and C7 G41D were in distal-mutational PTM impact (Fig. 6). The reference amino acid residues for C7 Q29R, C7 G41D, and C7 T756I were Q, G, T, and mutant amino acid residues reported were I, R, and D, respectively (Supplementary Table 2).

### Protein–protein interaction analysis

**Figure 7:**
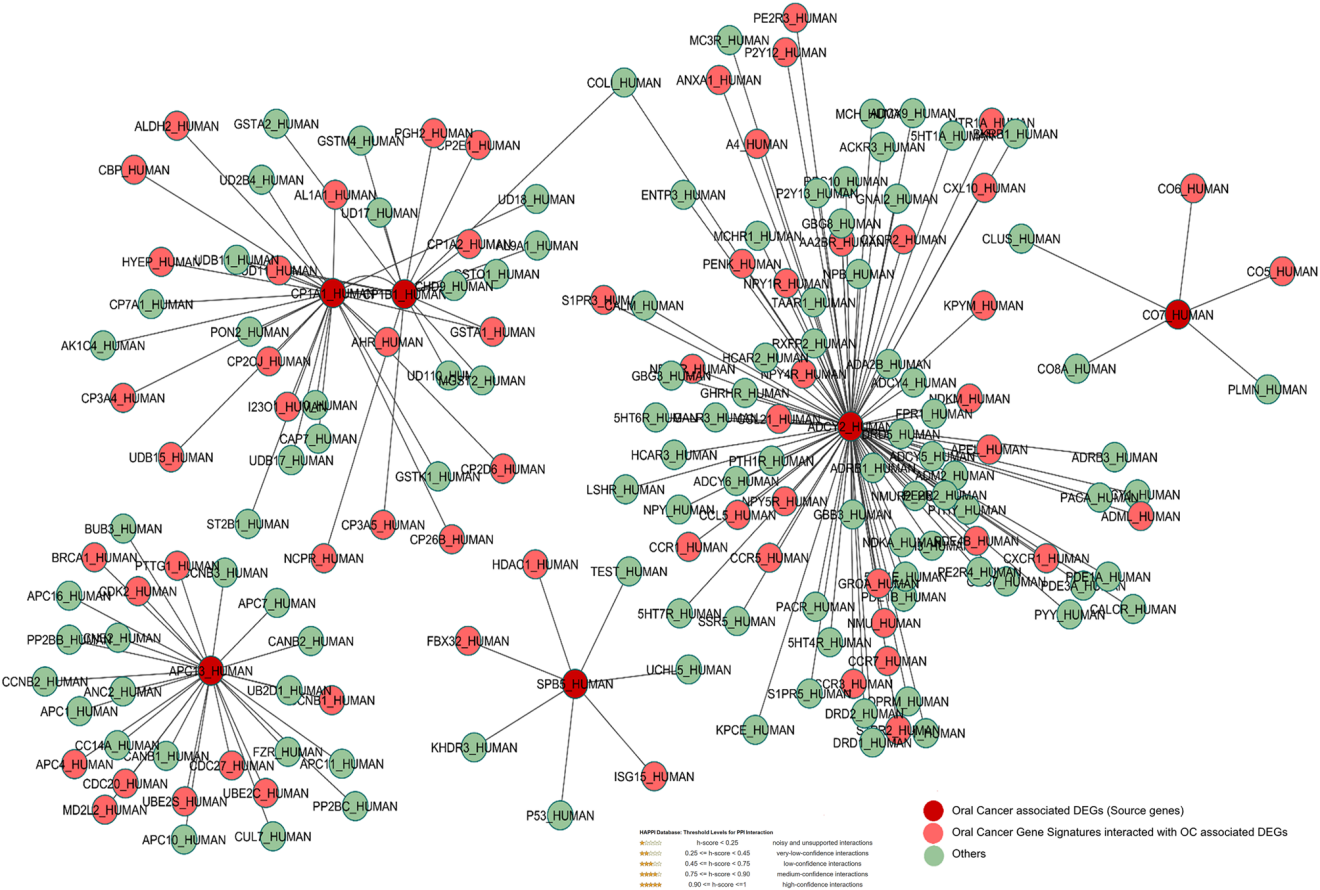
Protein–protein interaction of OC genes. Red nodes represent DEGs interacting with Pink nodes (target genes/gene signatures). High-confidence interactions of HAPPI database were selected in this network (the five stars are equivalent to high score (0.90–1).

We retrieved the related nodes and edges of all oral cancer associated DEGs from the HAPPI database to construct the integrated PPI-network (Fig. 7). This interaction analysis helped us to observe the potential functional interaction among OC related DEGs and other associated genes contributing to the disease phenotype. The seeder or source OC associated DEGs CYP1A1_HUMAN, CYP1B1_HUMAN, ADCY2_HUMAN, C7_HUMAN, SERPINB5_HUMAN, ANAPC13_HUMAN interact with the target genes including BRAC1_HUMAN, CO6_HUMAN, ISG15_HUMAN, S1PR3_HUMAN, and other essential proteins. The network topology shows a significant relationship between seeder and target genes. The identified proteins showed a significant association with disease development. The target proteins GST2_HUMAN and GSTK1_HUMAN play important role in phase-II carcinogen metabolism and interacted with source genes CYP1A1_HUMAN and CYP1B1_HUMAN. The target proteins including CO7_HUMAN, CLUS_HUMAN, CO6_HUMAN and CO5_HUMAN involved in laryngeal carcinoma, oral cavity squamous cell carcinoma and oropharyngeal carcinoma, respectively. Oral cancer ADCY2_HUMAN linked with KNG1_HUMAN as potential biomarkers. SPB5_HUMAN is interacted with P53_HUMAN, which is a potential tumor suppressor gene. We have observed that OC related genes APC13_HUMAN is interacting with CDK2_HUMAN, a prognostic indicator of oral cancer.

### Pathway modelling of OC associated DEGs

**Figure 8:**
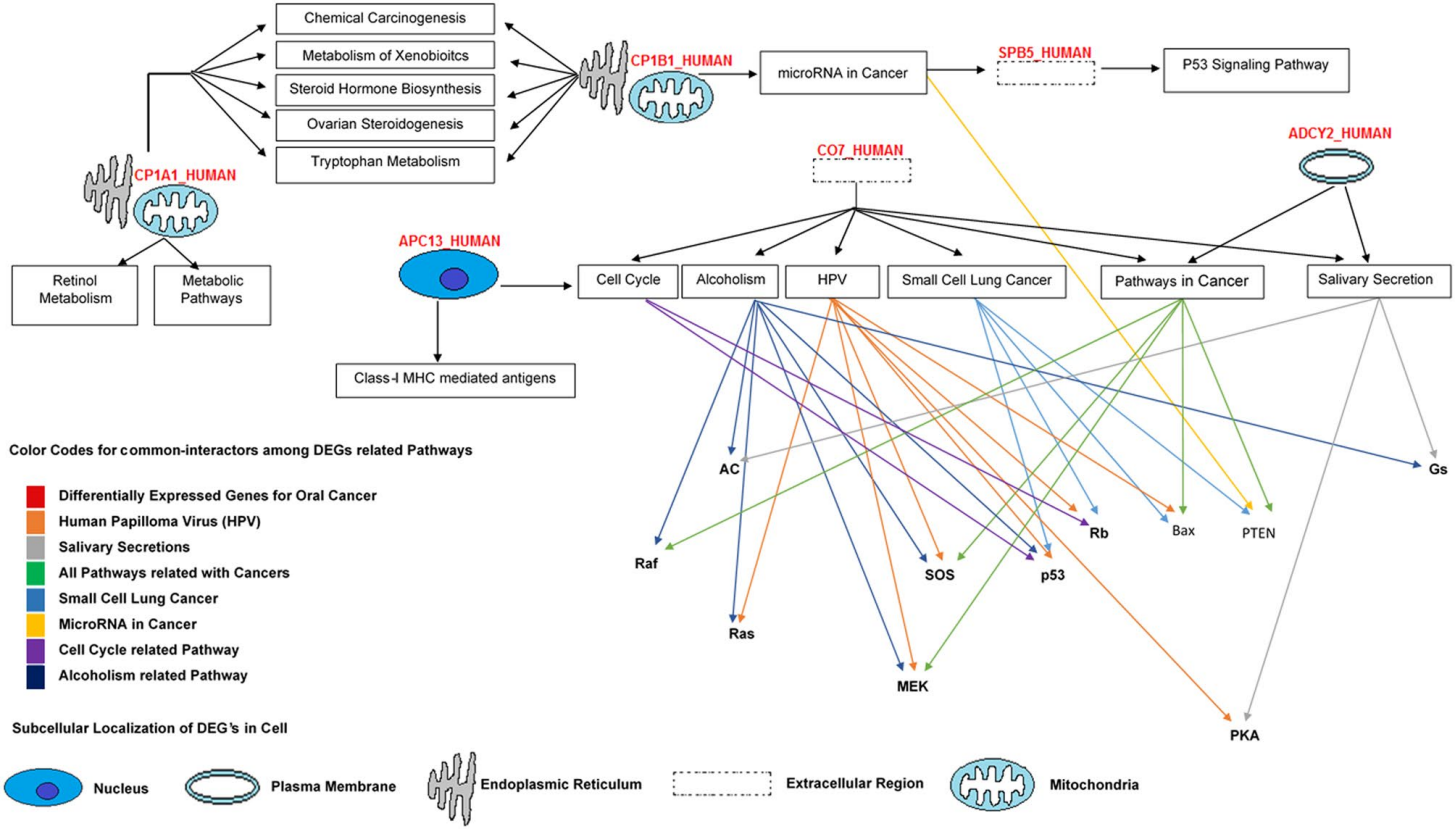
Pathway modeling for genome signaling and metabolic reconstruction revealed the pathological mechanism of oral cancer using KEGG and Wiki pathway databases.

Integrated pathways were modeled to observe the possible role of DEGs in pathophysiological mechanisms. Ras, p53, MEK, SOS, Rb, Bax, PTEN, and Raf are important interacting genes associated with the pathophysiological mechanism of oral cancer. We found that p53 signaling, microRNA signaling, salivary secretion, human papillomavirus, cell cycle, alcoholism, and xenobiotic metabolism-related pathways are interconnected in the progression and development of the disease (Fig. 8).

### Toxicogenomic analysis

**Figure 9:**
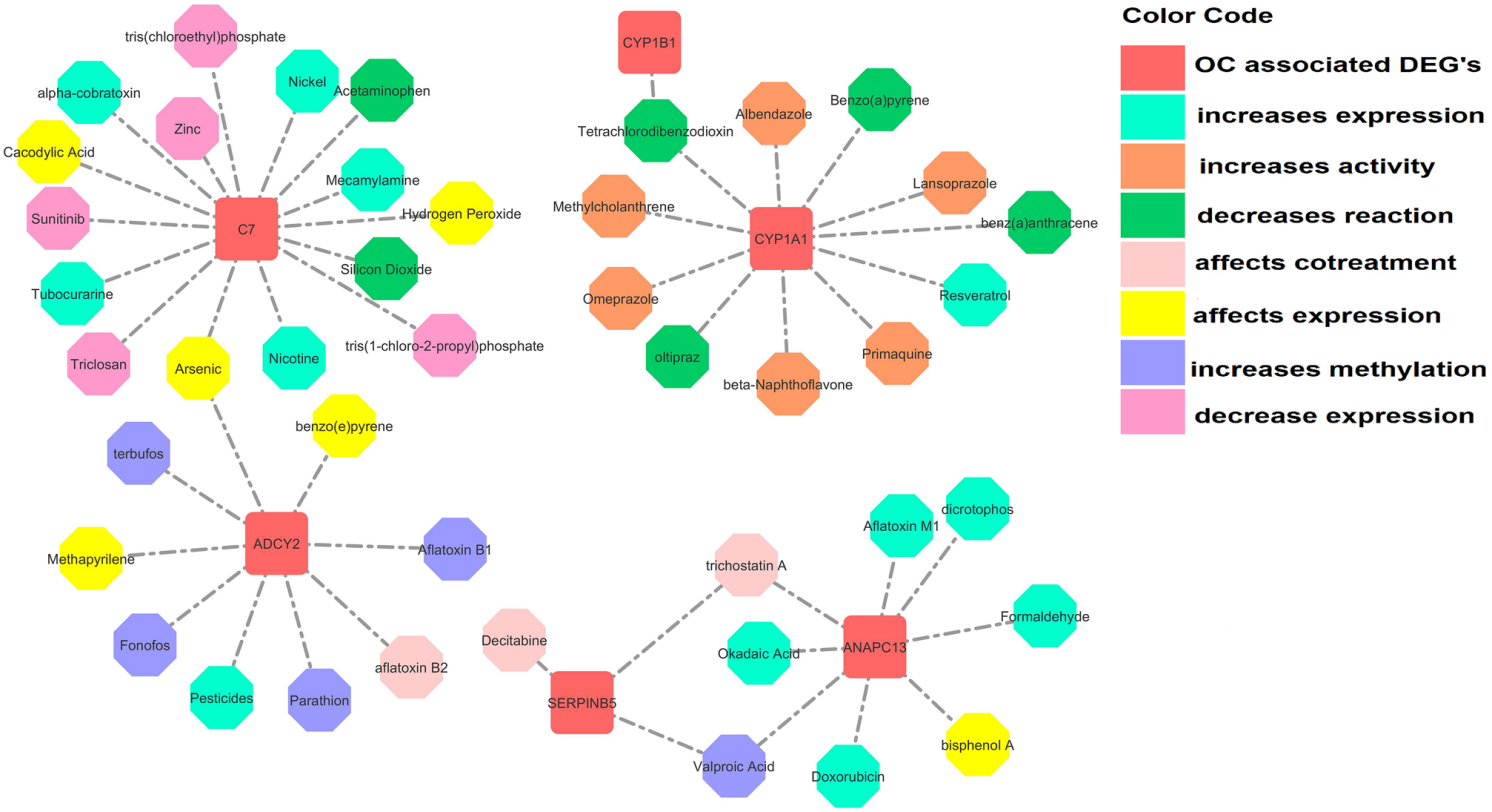
Toxicogenomic analysis of differentially expressed genes carried out by a comparative toxicogenomic database (CTD) helps to study the chemical-genome to phenome relationships.

The toxicogenomic analysis enabled us to explore chemical genotype–phenotype exposomic information that may lead to disease progression. OC associated DEGs were curated in terms of their activity and expression with different environmental chemicals. The data revealed the activity and expression of DEGs, which either increase or decrease the expression, increase or decrease towards gene activity at different cellular events. It may also affect the cotreatment expression leading to disease occurrence. It was revealed that the same chemical exposure may show different reactivity for different genes. In this case, benzo(a)pyrene increases the expression of CYP1A1 but it affects the reactivity of ADCY2. Methylcholanthrene, albendazole, primaquine increases CYP1A1 activity. Resveratrol and tetrachlorodibenzodioxin were found to affect the binding, decrease the reaction, and increase the CYP1A1 and CYP1B1 activity. Similarly, acetaminophen was found to increase the expression of C7, whereas alpha-cobra toxin may account for decrease reaction. Arsenic may affect C7 expression by increasing its abundance. While nickel was found to decrease C7 expression. However, decitabine was found to affect the cotreatment of SERPINB5 and decrease the gene reactivity with trichostatin A. It was observed that fonofos, methapyrilene, parathion increases and affects the ADCY2 reactivity at different cellular events while valproic acid, okadaic acid, doxorubicin, and bisphenol A decreases the ANAPC13 expression (Fig. 9).

### De novo prediction of regulatory motifs

Oral cancer-associated DEGs were used for de novo analysis to predict the regulatory motifs. The transcriptional factors include ARNT, AHR, CEBPA, CTCF, HNF1B, ELK4, TCF3, and NR2E3. The conservation cutoff is 0.40 with a matrix-score threshold of 85% were set as default parameters. The parameter settings standardize to analyze oPOSSUM-tool showed how the transcriptional factor controls its related targets (Table 5).

**Table 5:** Over-representation of oral cancer-related DEGs using oPOSSUM with 80% matrix score.

TF	Class	Family	Tax group	IC	Target gene hits	Target gene non-hits	Back-ground gene hits	Back-ground gene non-hits	Target TFBS hits	Target TFBS nucleotide rate	Back-ground TFBS hits	Back-ground TFBS nucleotide rate	Z-score	Fisher score
ESR1	Zinc-coordinating	Hormone-nuclear Receptor	Vertebrates	13.563	1	5	345	24,407	1	0.00319	355	0.000197	16.419	2.514
Arnt::Ahr	Zipper-Type	Helix-Loop-Helix	Vertebrates	9.532	5	1	13,597	11,155	23	0.022	66,086	0.011	8.273	1.815
CEBPA	Zipper-Type	Leucine Zipper	Vertebrates	8.712	5	1	12,828	11,924	18	0.0258	55,606	0.0139	8.01	2.06
CTCF	Zinc-coordinating	Beta Alpha-zinc finger	Vertebrates	17.205	2	4	3,236	21,516	2	0.00606	3,982	0.0021	6.703	1.718
HNF1B	Helix-Turn-Helix	Homeo	Vertebrates	16.821	3	3	4,107	20,645	3	0.00574	6,910	0.0023	5.543	2.787
ELK4	Winged Helix-Turn-Helix	Ets	Vertebrates	14.123	2	4	6,034	18,718	4	0.00574	9,342	0.00234	5.452	0.796
TAL1::TCF3	Zipper-Type	Helix-Loop-Helix	Vertebrates	14.07	2	4	7,491	17,261	5	0.00957	14,681	0.0049	5.21	0.535
Nr2e3	Zinc-coordinating	Hormone-nuclear Receptor	Vertebrates	12.028	4	2	6,354	18,398	5	0.00558	12,287	0.00239	5.045	3.187
Lhx3	Helix-Turn-Helix	Homeo	Vertebrates	16.354	2	4	5,806	18,946	4	0.00829	12,758	0.00461	4.212	0.846
Nkx2-5	Helix-Turn-Helix	Homeo	Vertebrates	8.27	6	0	16,973	7,779	43	0.048	197,210	0.0384	3.939	2.263
Arnt	Zipper-Type	Helix-Loop-Helix	Vertebrates	10.992	4	2	7,052	17,700	5	0.00478	13,904	0.00232	3.928	2.827
NFE2L2	Zipper-Type	Leucine Zipper	Vertebrates	14.394	2	4	5,635	19,117	3	0.00526	8,970	0.00274	3.696	0.886
ESR2	Zinc-coordinating	Hormone-nuclear Receptor	Vertebrates	13.618	1	5	2,219	22,533	1	0.00287	2,670	0.00134	3.155	0.842
Tal1::Gata1	Zipper-Type	Helix-Loop-Helix	Vertebrates	11.297	2	4	6,212	18,540	3	0.00861	11,200	0.0056	3.107	0.758
Evi1	Zinc-coordinating	BetaBetaAlpha-zinc finger	Vertebrates	17.909	1	5	1931	22,821	1	0.00223	2,457	0.000956	3.067	0.952
PLAG1	Zinc-coordinating	BetaBetaAlpha-zinc finger	Vertebrates	19.352	1	5	1971	22,781	1	0.00223	2,462	0.000958	3.059	0.936
MZF1_5-13	Zinc-coordinating	BetaBetaAlpha-zinc finger	Vertebrates	9.4	5	1	13,425	11,327	17	0.0271	77,649	0.0216	2.97	1.868
STAT1	Ig-fold	Stat	Vertebrates	13.119	2	4	6,394	18,358	3	0.00717	10,990	0.00458	2.949	0.722
Mycn	Zipper-Type	Helix-Loop-Helix	Vertebrates	11.104	3	3	8,332	16,420	5	0.00797	18,928	0.00526	2.883	1.12
Prrx2	Helix-Turn-Helix	Homeo	Vertebrates	9.063	4	2	15,063	9,689	27	0.0215	123,270	0.0171	2.636	0.576
FOXD1	Winged Helix-Turn-Helix	Forkhead	Vertebrates	11.926	6	0	13,087	11,665	14	0.0179	62,921	0.014	2.556	3.823
MEF2A	Other Alpha-Helix	MADS	Vertebrates	15.709	2	4	7,006	17,746	4	0.00638	15,631	0.00434	2.354	0.612
Gfi	Zinc-coordinating	BetaBetaAlpha-zinc finger	Vertebrates	9.47	4	2	13,731	11,021	14	0.0223	65,844	0.0183	2.332	0.796
FOXO3	Winged Helix-Turn-Helix	Forkhead	Vertebrates	11.734	5	1	13,611	11,141	15	0.0191	69,410	0.0154	2.328	1.811

### MiRNAs targets for oral cancer-specific genes

miRDB database helped us to predict the miRNA targets based on the algorithms. We found the reliable score (> 80) of oral cancer-specific miRNAs. Mainly miRNAs including hsa-miR-4786-5p, hsa-miR-4282, hsa-miR-2052, hsa-miR-216a-3p, hsa-miR-3148, and hsa-let-7f-1-3p targets were predicted for CYP1A1, CYP1B1, C7, ADCY2, SERPINB5 and ANAPC13 genes respectively. The functional dysregulation of these genes may lead to disease progression. The predicted target scores, total miRNA hits, seed-binding locations, and 3′-UTR length of DEGs were analyzed (Table 6).

**Table 6:** Prediction of gene specific MiRNA-targets associated with oral cancer.

Serial no	Gene symbol	Gene description	*Target score	microRNA name	Total hits	miRNA sequence	Seed location	3′-UTR length
1	CYP1A1	cytochrome P450 family 1 subfamily A member 1	90	hsa-miR-4786-5p	35	UGAGACCAGGACUGGAUGCACC	735	966
2	CYP1B1	cytochrome P450 family 1 subfamily B member 1	98	hsa-miR-4282	141	UAAAAUUUGCAUCCAGGA	19, 704, 1,092, 1,235, 1,246, 1753, 2028, 2,137	3,125
3	C7	complement C7	97	hsa-miR-2052	84	UGUUUUGAUAACAGUAAUGU	1,197, 1,203, 1,311	3,073
4	ADCY2	adenylate cyclase 2	96	hsa-miR-216a-3p	142	UCACAGUGGUCUCUGGGAUUAU	190, 1575, 1641, 2,853	3,210
5	SERPINB5	serpin family B member 5	98	hsa-miR-3148	64	UGGAAAAAACUGGUGUGUGCUU	169, 196, 1,323	1,363
6	ANAPC13	anaphase promoting complex subunit 13	89	hsa-let-7f-1-3p	61	CUAUACAAUCUAUUGCCUUCCC	699	903

*Highly reliable score—> 80, least reliable score—< 50.

### Drug-gene interaction analysis

**Figure 10:**
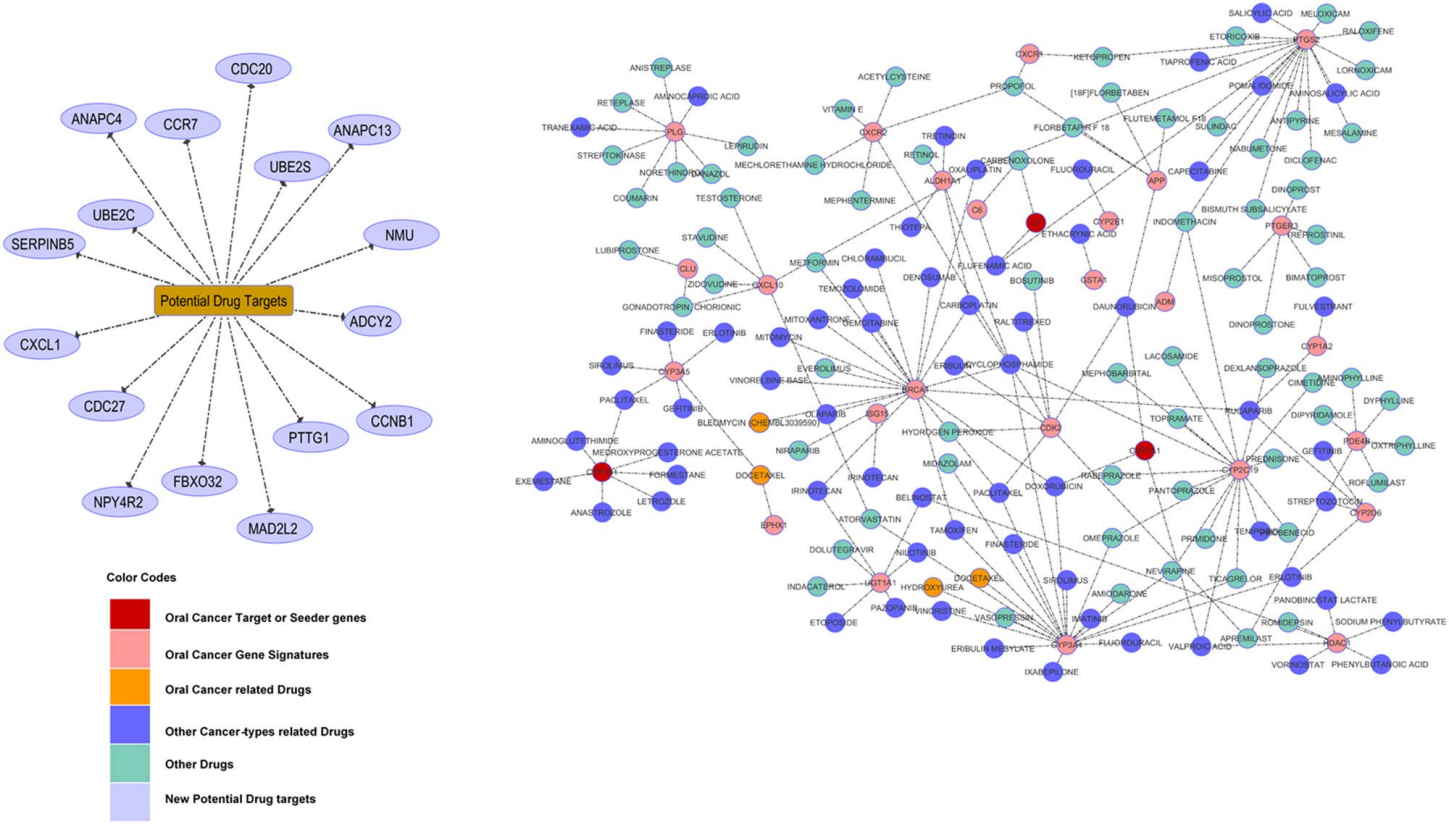
The drug–gene network was constructed between the FDA approved drugs and target genes. A broken line indicates the interaction between known drugs while solid line represents the novel drug targets. Anticancer drugs were retrieved from drug B+ ank.

The toxicogenomic approach was used to investigate the drug-genes interaction to explore available treatments. The genes that interact with anticancer drugs are docetaxel, hydroxyurea, bleomycin, daunorubicin, lansoprazole, doxorubicin, liothyronine sodium, risperidone using DGIdb database. We identified sixteen proteins as potential alternative drug targets including CXCL1, FBXO32, PTTG1, CCNB1, ADCY2, NMU, ANAPC13 and others (Fig. 10). The dysregulation of these proteins may affect the normal expression level and could be a potential part of therapeutic strategies.

## Discussion

This study focused on the genetic expression and functional enrichment of genetic variants of oral cancer. The six most significantly OC associated genes (*CYP1A1*, *CYP1B1*, *SERPINB5*, *ANAPC13*, *ADCY2*, *C7*) found through a differential analysis were consider as seeder or source genes. This analysis provides us a list of new genes aberrantly expressed in oral cancer including *SERPINB5*, *ANAPC13*, *ADCY2*. We have investigated differential expression between cases and controls of cDNA datasets at cellular level in oral tissues and found the possible association of these genes in oral cancer. We can get more information about the mechanism of human genetic disorder through microarray studies. The expression profiling of these important genes shows obvious differences between cases and controls. Some DEGs were found upregulated while other downregulated in this analysis. These genes are abnormally expressed to affect physiological functions including cellular signaling, replication, mitotic division, and programmed cell death. We have observed that our differentially expressed genes are associated with the cancer pathways including biological oxidations, metabolism, adenylate cyclase-activating pathway, xenobiotics, G alpha signaling events, transcriptional targets of TAp63 isoforms, p53, and IFN-gamma pathway revealed the biological significance of these genes specifically for oral cancer progression. CDC20_HUMAN, HDAC1_HUMAN, CXL10_HUMAN interacting with source genes are potential drug targets^13–15^. The inherent mutations are reported for genes that encode drug-metabolizing enzymes. Such somatic gene mutations are induced chemically that play a vital role in cell differentiation and growth^16^. The sequencing investigations not only characterizes the genomics but also revealed thousands of SNVs (single nucleotide variants), the alterations in copy number along with many types of genetic variations. Such genomic to phenomic association identification, their molecular-level mechanisms, disease-related variants along with cancer-derived mutations are the current challenges in the biomedical research^17,18^. Deciphering inter-individual genetic variation is the latest trend in personal genomic era investigations. The interpretation of genomic to proteomic information may integrate the impact of mutations on cellular system-level investigations in the future with a higher magnitude^19^. Human proteomic data analysis uses PTMs to interpret disease mutations and inter-individual genetic variations. PTMs being important regulators of protein function and signaling pathways facilitate the missense mutational analysis investigations^19^.

We have observed that *CYP1A1 *belongs to potential and well-preserved phase-I xenobiotic metabolizing gene family which is involved in the activation of procarcinogens. The *CYP1A1 *enzyme is highly associated with increased risk of tumors in the oral cavity, bronchial and laryngeal regions in smokers^4^. Similarly, the association of *CYP1B1* has found in many cancer types^20^. Many investigations are reported about the substantial link of HNC with *CYP1A1* and *CYP1B1*^16,21^.

*SERPINB5* belongs to serpin encoding serine protease which plays a vital role in tumor metastasis^22,23^. It is a tumor suppressor in epithelial cells and can suppress cancerous cell invasion and their metastasizing in surrounding tissues^24^. The paradoxical expression of *SERPINB5* has been observed in various types of tumor^25–28^. We have seen a highly significant association between *SERPINB5* expression and oral carcinoma^29^. *ADCY2* is a membrane-associated enzyme which converts adenosine-5′-triphosphate (ATP) into 3′,5′-adenosine monophosphate (cyclic AMP/cAMP) and pyrophosphate^30^ involving in the regulation of cAMP synthesis^31^. This gene catalyzes the signaling molecule cAMP through G-protein beta as well as gamma subunit signaling^32,33^. Therefore, changes in expression patterns of the gene are mediated through down-streaming of the signaling cascades muscarinic acetylcholine receptors which increases IL6 production. The high regulation of the gene is observed in G-proteins, calcium, calmodulin, pyrophosphate, and post-translational modifications. The signaling pathways include RET signaling, Oocyte meiosis, calcium, and chemokine signaling pathways^34–36^. Aberrant methylation of *ADCY2* is observed in colorectal, prostate cancer^35–37^ and urinary bladder cancer^38–40^. It has been studied that *ANAPC13*^41^ is a large-sized ubiquitin ligase that controls the cell cycle progression^42^ and involved at early steps of malignancy in tumor cells^43^. Similarly, *C7* (complement component-7), the terminal component for complement cascade and as a cytolytic effector for complement system, lyses transformed malignant cells^44–51^. The integration of chemical- gene interaction revealed different environmental chemical exposure to disease progression^52,53^. This analysis helped to reveal the mechanism of action between the chemical and the related gene products and their effect on human disease influence by environmental exposure^53–56^.

The PPI network predicted the important association of these genes with disease. These genes have a potential role in xenobiotic metabolism, tumor progression, suppression, cell cycle, HPV (human papilloma virus), alcoholism, and microRNA signaling pathway^30,40,48,49,51,57–60^. The transcriptomic analysis showed expressive transcription factors like JUND, FOXO, STAT1. We found the role of these genes in metabolism of xenobiotics, p53 signaling, salivary secretion, class-I MHC mediated antigens and microRNA cancer pathways. The miRNAs regulate post-transcriptional and translational events and expressional dysregulation in these molecules leads towards the progression of many diseases^61–63^. Therefore, the reliable miRNA target prediction is crucial for the functional annotation of miRNAs^64,65^.

Recent reports proved that the drug-gene network enables us to understand not only the disease pathophysiology but also important in drug designing or new drug target identification or establishing novel biomedical linkages. More importantly, this network proposed many testable assumptions with the potential of great success, though the real achievement can only be justified by experimental studies.

## Conclusion

Our simulation-based systems-level hypothesis is comprehensive and effective to sort out the disease-specific genetic variants from cDNA datasets repositories. Therefore, this approach will support understanding the genetic basis of complex phenotypes including cellular replication, protein signaling, mitotic division, and programmed cell death. Based on genomic to phenomic investigations, we have found new genes including *ADCY2*, *SERPINB5*, and *ANAPC13* linked with oral cancer that could be potential diagnostic or drug targets. These source genes are clearly interacting with other essential genes affecting cell cycle and apoptosis causing carcinogenesis. These findings can provide a valuable framework for developing new therapeutic strategies against oral cancer.

## Methods

### Accession to cDNA datasets

**Figure 11:**
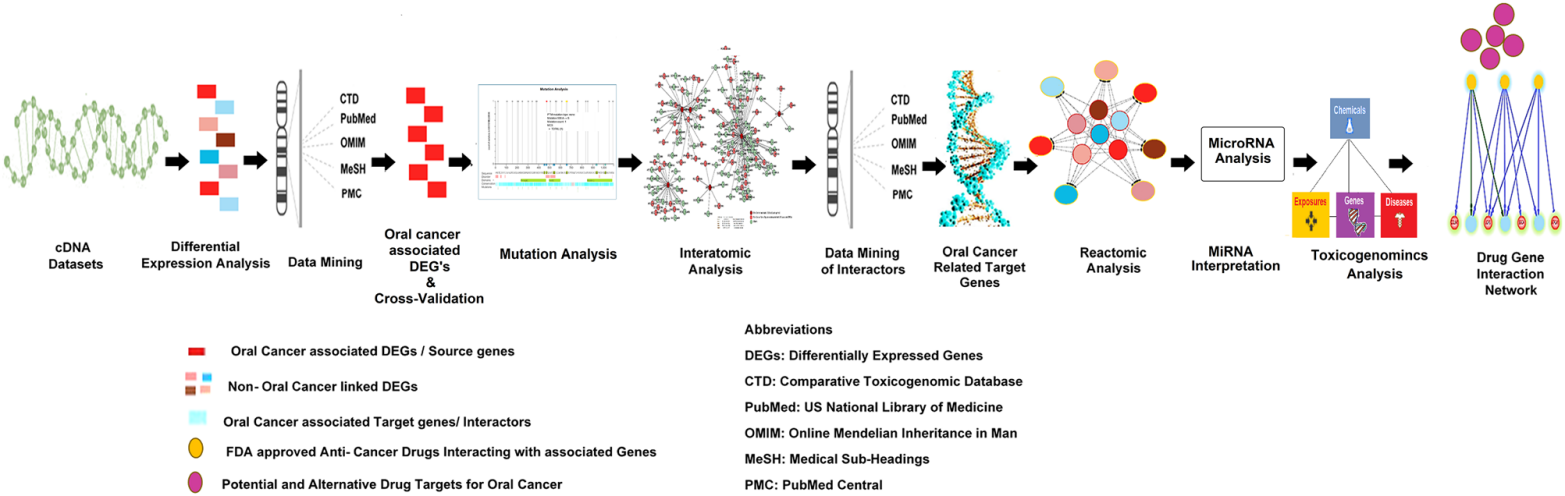
The steps have been integrated in basic framework of our study.

5.2 We downloaded cDNA datasets related to oral cancer from the Gene Expression Omnibus database (GEO) NCBI. The comprehensive framework has been illustrated in Fig. 11 using tools, online servers, and software (Table 7).

**Table 7:** Databases and tools used during this meta-analysis.

Databases/oftware/tools	Accessibility	Utility	References
STRING database version 11	https://string-db.org/	For known and predicted protein/COGs interaction	74
National Center for Biotechnology Information (NCBI)	https://ncbi.nlm.nih.gov	Biomedical and genomic information source	–
Cytoscape version 3.6.0	https://www.cytoscape.org/	For network analysis and visualization	31
DAVID Bioinformatics tool 6.8	https://david.abcc.ncifcrf.gov	Gene ontology/ Functional Annotation tool	75
Uniprot	https://www.uniprot.org/	Resource of protein functional information	76
Kyoto Encyclopedia of Genes and Genomes (KEGG)	https://www.genome.jp/	Pathways analysis and comparison	77
FunRich version 3	www.funrich.org/	Enrichment analysis	24
R version 3.3.3	https://www.r-project.org/	Statistical computing/data mining	14–16
Opossum Version 3.0	https://opossum.cisreg.ca/oPOSSUM3/	Single site analysis	78
Wiki-Pathways	https://www.wikipathways.org/index.php/WikiPathways	Pathways analysis	79
Path-Visio 3.3.0	https://www.pathvisio.org/	pathway analysis and drawing software	34
Comparative Toxicogenomics Database CTD	https://ctdbase.org/	gene–disease relationships	80
CIMminer	https://discover.nci.nih.gov/cimminer/home.do	Cluster analysis	25–26
HAPPI version 2,0	https://discovery.informatics.uab.edu/HAPPI/	Protein–protein interaction	30

### Normalization and differential analysis

These datasets were analyzed in identifiable format to easily access pheno-data files and missing values were imputed^66^. R software version 3.3.3 and Bioconductor packages were used in computational analysis. The normalization and quality control analysis was performed to preprocess the information available by ArrayQuality^67–69^. The background and normalization were aligned by using Robust Multi-Array Analysis (RMA) to detect the PM (perfect matches) and the MM (mismatches) to impute the values for statistical analysis^70^. RMA is the widely used preprocessing algorithm applied for background correction to remove local artifacts^67^.

$$ PM_{ijk} = BG_{ijk} + S_{ijk} $$

where *PM* indicates a perfect match, Background by *BG* and non-specific binding (*S*); *ijk* is the signal for probe *j* of probe set *k* on array *i*.

$$\begin{aligned} &BG\left( {PM_{ijk} } \right) = E\left[ {S_{ijk} |PM_{ijk} } \right] > 0\\ &S_{ijk} \sim Exp(\lambda_{ijk} )\quad BG_{ijk} \sim N(\beta_{i} ,\sigma^{2} ). \end{aligned} $$

The perfect match involves the combined signals of background (BG) and expression (E). The "ArrayQualityMetrics" software was used to evaluate the quality of dataset that is normalized to each genke's median expression level^67,68,71^. The gene–gene covariance matrix of each data set was calculated across all arrays while ignoring the missing values. The transformation formula is:

$$ X_{norm} = F2^{{^{ - 1} }} \left( {F1\left( x \right)} \right) $$

where *F*1 and *F*2 represents distribution functions of the actual and reference chips.

To get a description of intensities, we used the RMA-algorithm to measure averages between probes in a sample set. During this analysis RNA quality was evaluated in samples. The RNA degradation analysis was performed by using AffyRNAdeg, summary AffyRNAdeg, and plot AffyRNAdeg packages^72^. The DEGs were observed using the LIMMA package, that process the information based on modified statistics which is proportional to sample variance offsets. The LIMMA package measured the duplicate spots and quality weights. The statistical analysis was performed to categorize the genes based on the significant cutoffs values logFC greater than 1, FDR less than 0.05, AEL ≥ 40% and *p* value ≤ 0.05^73^.

### K-fold cross-validation

We used K-Fold Cross-validation and Bootstrap test to estimate accuracy in the differential analysis^74^ and this approach has the advantage that all the samples in the dataset can ultimately be used for both training and research. This technique is usually easier to calculate estimated average error and has been used to validate the shortlisted differentially expressed genes using the 'Boot' package of Bioconductor. Boots trapping is used effectively in molecular analysis to correct biases^75^. In such cases, we applied the generalized linear Gaussian models and used the 'cv.glm' method to test the k-fold cross-validation. It estimates the true error as the average rate error:

$$ E = 1/K\mathop \sum \limits_{i = K}^{K} Ei $$

Leave-one-out-cross-validation (LOOCV) continued to trail the Gaussian rule. The LOOCV approach is instinctively termed as the test set is left out and the rest of the data is used as the training-set^75^. We used N − 1 subsets for training and the rest for testing. Increasing the number of folds would make the bias of the true error rate estimator low and valid^75,76^.

The true error is assessed as the average error rate on test cases:

$$ E = 1/N\mathop \sum \limits_{i = K}^{N} Ei $$

### Disease-gene curation of differentially expressed genes (DEGs)

The text mining is important in biomedical research to extract useful information^77^. This analysis is designed to identify the most significant DEGs, all 30 genes from 21 datasets were curated from the DAVID database to retrieve their gene symbol, gene name, Uniprot_ID. These genes were curated using Comparative Toxicogenomics Database (CTD), Online Mendelian Inheritance in Man (OMIM), PubMed and MeSH databases to observe their role in oral cancer. This screening further shortlisted the significant DEGs^78^.

### Enrichment and cluster analysis

The biological functions of the genes help us to understand the cellular level signaling network. We performed enrichment analysis using the DAVID tool^78–80^. FunRich tool was used to observe the biological functions of oral cancer-related DEGs at molecular level^81^. The list of DEGs were analyzed for their *p*- and FDR values^79^. For cluster analysis, gene expression values of cases and controls of each dataset were studied to observe genetic variations and expression profiling using One Matrix CIMminer tool^82,83^.

### Mutation analysis

Mutations resulting from cancer and the inherited-disease process can be understood to decode the genetic variation by associations of genotype-phenotype. The human genome contains thousands of SNVs (single nucleotide variants) and many are known for the progression of the disease. Approximately 21% of amino acid substitutions are known to be associated with disease-progression in correspondence with missense single nucleotide variants located at PTM protein sites (post-translation modifications). The chemical modification of the amino acid thus basically extends the functionality of the associated protein^19^.

Mutation of differentially expressed genes were analyzed using online ActiveDriverDB database^19^. The needle plot mutations analysis provides a visual overview of the position, frequency, and functional significance of all identified mutations in our DEGs. PTM sites with all mutations and the predicted disordered region of protein sequences were observed. Placing the pins corresponds to the position along the sequence of the genes and protein, whereas the related mutation effect and PTM are explained in the figure legend.

### Protein–protein interaction

The biological functions are mainly carried out by protein–protein interactions^30,84^. The interaction of proteins reveals that each protein interacts with one or more genes related to their molecular functions^85^. The biological networks indicate altered activity in normal or disease conditions. This gene-network aims to identify potentially OC associated gene signatures whose dysfunction directly contributes to disease phenotype are functionally associated. The gene signatures related to each source protein was measured. Human Annotated and Predicted Protein Interaction (HAPPI) and String databases were used to analyze gene–gene/protein–protein interactions of microarray dataset DEGs^86^. This database annotates and mine comprehensive physical as well as genetic mapping and includes experimentally validated data to simulate biological networks. We have mentioned the threshold for PPI network from HAPPI database. We used high-confidence interactions in our network (the five stars are equivalent to high score (0.90–1). The role and association of these source and target genes in oral cancer were evaluated from Cancer Genetics Web, National Cancer and OMIM database. The molecular networks were visualized by Cytoscape software (version 3.6.0)^87^. The Cytoscape Network Analyzer calculates topological properties of networks. The degree of annotation between the gene and disease is categorized by nodes in the network.

### Pathway analysis of oral cancer linked genes

Reactomic analysis enable us to explore all metabolic networks of DEGs regarding their molecular mechanism. We analyzed these pathways to inter-connect DEGs to show the pathological mechanism of oral cancer. The KEGG and Wiki pathways databases were used to map target genes^88,89^. PathVisio tool was used to reconstruct the pathway model for understanding system-level analysis^90^.

### Toxicogenomic analysis

The toxicogenomic analysis is carried out by a comparative toxicogenomic database (CTD) to retrieve exposome data. The exposome data helps investigate chemical-genome to phenome relationships to interpret the functional pathway cellular signaling-mechanism towards disease progression influenced by environmental exposures. It provides information regarding chemical-gene/protein and disease interactions which may reveal the particular gene-activity or expression regarding gene-disease connections. The curation of environment-disease exposure helps to analyze the available toxicogenomic information^55^.

### Prediction of regulatory motifs

Cancer has a complex mechanism that can be explored by understanding the biological functions at transcription and post-transcriptional level. oPOSSUM version 3.0 was used to analyze promoter region target motifs like transcription factor binding sites (TFBS) or the overexpression of target matrices^91,92^. This information helps to understand the functional role of gene targets and eventually gene ontology^93^.

### Prediction of oral cancer-associated miRNA targets

Numerous genes are involved in the biological signaling cascade. These cascades are influenced through small noncoding RNAs as post-transcriptional regulators, known as microRNAs (miRNAs). The function and expression of miRNA play a significant role in understanding gene etiology^5,93^. miRNA target prediction helps to explore the functional and molecular annotation of disease-specific DEG’s^5,94^. Therefore, oral cancer associated DEG’s miRNA targets were predicted by miRDB, an online database for functional microRNA target prediction. The target prediction data involves specie specific 3′-UTR sequences, 3′-UTR region length, miRNA seed binding-sites, miRNA-candidate target pairs along with target prediction scores, miRNA-target sequences, and other important description^95,96^. The MiRNA target predictive score is ranked and > 80 was considered as a reliable score^95,96^.

### Drug-gene interaction analysis

In our study, the drug-gene network analysis was performed to correlate our shortlisted DEGs with FDA approved commercially available anti-cancer drugs. CTD database was used to investigate the relationship between the chemical and disease at default parameters. In this analysis, DEGs were directly linked to anticancer drugs. All drugs, used in this interaction, were verified through the Drug Bank database to check approval status by the FDA.

## Supplementary information

Supplementary information

## Data Availability

All the other data that support the findings of this study are available from the corresponding author upon request.
