# Supplementary material for: Systems-level differential gene expression analysis reveals new genetic variants of oral cancer
Source: Sci Rep. 2020 Sep 4;10:14667. doi: 10.1038/s41598-020-71346-7 (PMC7473858; doi:10.1038/s41598-020-71346-7)
Supplement: Supplementary file 1 — Supplementary information [file 41598_2020_71346_MOESM1_ESM.pdf]

Systems-Level Differential Gene Expression Analysis Reveals New Genetic Variants of Oral Cancer

Syeda Zahra Abbas, Muhammad Imran Qadir, Syed Aun Muhammad
Institute of Molecular Biology and Biotechnology, Bahauddin Zakariya University Multan, Pakistan

Supplementary Table 1: Data Mining of 30-Differentially Expressed Genes (DEGs).

The text mining is important in biomedical research to extract useful information.

This analysis is designed to identify the most significant DEGs.

All 30 genes from 21 datasets were curated from DAVID database to retrieve their gene symbol, gene name, Uniprot_ID.

These genes were curated using Comparative Toxicogenomics Database (CTD), Online Mendelian Inheritance in Man (OMIM), PubMed and MeSH, PMC databases to observe their role in oral cancer.

This screening further shortlisted the significant DEGs.

To	Species	David Gene Name	KEGG	WIKI Pathway	PubMed Count	PMC	MeSH	OMIM	CTD	Reason of Selection / Rejection
ENSG00000109572	Homo sapiens	chloride voltage-gated channel 3(CLCN3)	No Hits	Yes	0	30	0	0	8	No Oral or Cancer Specific
ENSG00000164692	Homo sapiens	collagen type I alpha 2 chain (COL1A2)	No Hits	Yes	105	3111	0	2	8	No Oral or Cancer Specific
ENSG00000143226	Homo sapiens	Fc fragment of IgG receptor IIa (FCGR2A)	Yes	Yes	3	203	0	2	8	No Oral or Cancer Specific
ENSG00000035862	Homo sapiens	TIMP metallopeptidase inhibitor 2(TIMP2)	No Hits	Yes	84	703	0	0	8	No Oral or Cancer Specific
ENSG00000186847	Homo sapiens	keratin 14(KRT14)	Yes	Yes	20	411	0	0	8	No Oral or Cancer Specific
ENSG00000112319	Homo sapiens	EYA transcriptional coactivator and phosphatase 4(EYA4)	No Hits	Yes	1	72	0	0	8	No Oral or Cancer Specific
ENSG00000140465	Homo sapiens	cytochrome P450 family 1 subfamily A member 1(CYP1A1)	Yes	Yes	175	2797	0	3	25	Oral Cancer Specific
ENSG00000128739	Homo sapiens	small nuclear ribonucleoprotein polypeptide N(SNRPN)	No Hits	Yes	2	276	0	1	8	No Oral or Cancer Specific

ENSG00000111405	Homo sapiens	endonuclease, poly(U) specific (ENDOU)	No Hits	No Hits	19	41	0	0	43	No Hits
ENSG00000149573	Homo sapiens	myelin protein zero like 2(MPZL2)	No Hits	No Hits	0	17	0	0	8	No Hits
ENSG00000198363	Homo sapiens	aspartate beta-hydroxylase (ASPH)	No Hits	Yes	1	61	0	0	8	No Oral or Cancer Specific
ENSG00000104177	Homo sapiens	myelin expression factor 2 (MYEF2)	No Hits	Yes	0	8	0	0	8	No Oral or Cancer Specific
ENSG00000108244	Homo sapiens	keratin 23(KRT23)	Yes	No Hits	0	32	0	0	8	No Oral or Cancer Specific
ENSG00000138061	Homo sapiens	cytochrome P450 family 1 subfamily B member 1(CYP1B1)	Yes	Yes	60	1476	0	0	8	Oral Cancer Specific
ENSG00000262406	Homo sapiens	matrix metallopeptidase 12 (MMP12)	No Hits	Yes	11	436	0	2	8	No Oral or Cancer Specific
ENSG00000071794	Homo sapiens	helicase like transcription factor (HLTF)	No Hits	Yes	0	64	0	0	8	No Oral Specific
ENSG00000075391	Homo sapiens	RAS protein activator like 2(RASAL2)	Yes	Yes	0	40	0	0	8	No Oral or Cancer Specific
ENSG00000078295	Homo sapiens	adenylate cyclase 2(ADCY2)	Yes	Yes	2	41	0	0	8	Oral Cancer Specific
ENSG00000135919	Homo sapiens	serpin family E member 2(SERPINE2)	No Hits	No Hits	5	110	0	0	8	No Oral or Cancer Specific
ENSG00000206075	Homo sapiens	serpin family B member 5(SERPINB5)	Yes	No Hits	2	153	0	0	8	Oral Cancer Specific
ENSG00000101557	Homo sapiens	ubiquitin specific peptidase 14(USP14)	No Hits	Yes	1	155	0	0	8	No Oral or Cancer Specific
ENSG00000188021	Homo sapiens	Ubiquilin 2(UBQLN2)	Yes	Yes	0	56	0	0	8	No Oral or Cancer Specific
ENSG00000069974	Homo sapiens	RAB27A, member RAS oncogene family (RAB27A)	No Hits	Yes	3	367	0	0	8	No Oral or Cancer Specific
ENSG00000138061	Homo sapiens	cytochrome P450 family 1 subfamily B member 1(CYP1B1)	Yes	Yes	60	1476	0	0	8	Oral Cancer Specific
ENSG00000114698	Homo sapiens	phospholipid scramblase 4(PLSCR4)	No Hits	No Hits	0	13	0	0	8	No Hits
ENSG00000133110	Homo sapiens	Periostin (POSTN)	Yes	Yes	15	378	0	0	8	No Oral or Cancer Specific
ENSG00000124783	Homo sapiens	signal sequence receptor subunit 1 (SSR1)	Yes	Yes	1	40	0	0	8	No Oral or Cancer Specific
ENSG00000175414	Homo sapiens	ADP ribosylation factor like GTPase 10 (ARL10)	No Hits	Yes	0	3	0	0	8	No Oral or Cancer Specific
ENSG00000112936	Homo sapiens	complement C7(C7)	Yes	Yes	19	1965	0	1	8	Oral Cancer Specific
ENSG00000129055	Homo sapiens	anaphase promoting complex subunit 13 (ANAPC13)	Yes	Yes	1	10	5	0	9	Oral Cancer Specific

Supplementary Table 2: Mutational Visualization plot interpretation of each interacted mutation

Mutation	Position of mutation in Protein	Reference amino acid residue in protein	Mutated amino acid residue in protein	Count	Impact on closest PTM site	Number of adjacent PTMs affected	Affected site(s)
ADCY2 D24V	24	D	V	1	none	0	
ADCY2 R107H	107	R	H	1	none	0	
ADCY2 D157E	157	D	E	1	none	0	
ADCY2 G205A	205	G	A	1	none	0	
ADCY2 M212L	212	M	L	1	none	0	
ADCY2 P249R	249	P	R	1	none	0	
ADCY2 A303E	303	A	E	1	none	0	
ADCY2 C340Y	340	C	Y	1	none	0	
ADCY2 G377R	377	G	R	1	none	0	
ADCY2 R383C	383	R	C	1	none	0	
ADCY2 Q474R	474	Q	R	1	network-rewiring	1	472S
ADCY2 A508S	508	A	S	1	none	0	
ADCY2 R556S	556	R	S	1	none	0	
ADCY2 G563R	563	G	R	1	none	0	
ADCY2 I614N	614	I	N	1	none	0	
ADCY2 S655R	655	S	R	1	distal	1	659S
ADCY2 L661V	661	L	V	1	proximal	1	659S
ADCY2 E760Q	760	E	Q	1	none	0	
ADCY2 E760K	760	E	K	1	none	0	
ADCY2 N836K	836	N	K	1	none	0	
ADCY2 R853H	853	R	H	1	none	0	
ADCY2 G950C	950	G	C	1	none	0	
ADCY2 Q1051H	1051	Q	H	1	none	0	
ADCY2 Q1086K	1086	Q	K	1	none	0	
C7 Q29R	29	Q	R	1	distal	1	36W
C7 G41D	41	G	D	1	distal	1	36W
C7 P133H	133	P	H	1	none	0	
C7 E549V	549	E	V	1	none	0	
C7 F671C	671	F	C	1	none	0	
C7 H746Y	746	H	Y	1	none	0	
C7 T756I	756	T	I	1	motif-changing	1	754N

C7 P766T	766	P	T	1	none	0	
C7 S768L	768	S	L	1	none	0	
C7 A822V	822	A	V	1	none	0	
CYP1B1 I87S	87	I	S	1	none	0	
CYP1B1 Q479H	479	Q	H	1	none	0	
CYP1B1 T510I	510	T	I	1	none	0	
CYP1A1 S80C	80	S	C	1	none	0	
SERPINB5 S9L	9	S	L	1	direct	1	9S
SERPINB5 G55V	55	G	V	1	distal	1	50T
SERPINB5 V57L	57	V	L	1	distal	2	50T 64K
SERPINB5 H59D	59	H	D	1	distal	1	64K
SERPINB5 G69R	69	G	R	1	distal	1	64K
SERPINB5 D76N	76	D	N	1	distal	1	79K
SERPINB5 R91W	91	R	W	1	proximal	3	85S 93Y 97S
SERPINB5 D95N	95	D	N	1	proximal	3	93Y 97S 102T
SERPINB5 T128M	128	T	M	1	proximal	2	129K 135S
SERPINB5 I136T	136	I	T	1	proximal	3	129K 135S 137K
SERPINB5 D138Y	138	D	Y	1	proximal	2	135S 137K
SERPINB5 D155N	155	D	N	1	proximal	1	157T
SERPINB5 A165T	165	A	T	1	none	0	
SERPINB5 G169A	169	G	A	1	none	0	
SERPINB5 M172I	172	M	I	1	none	0	
SERPINB5 F175S	175	F	S	1	none	0	
SERPINB5 P194A	194	P	A	1	none	0	
SERPINB5 S211N	211	S	N	1	none	0	
SERPINB5 E218D	218	E	D	1	none	0	
SERPINB5 K224R	224	K	R	2	none	0	
SERPINB5 E328Q	328	E	Q	1	none	0	
SERPINB5 L348F	348	L	F	1	distal	1	345K
SERPINB5 I358T	358	I	T	1	proximal	1	356Y
SERPINB5 N361K	361	N	K	1	distal	1	356Y
ANAPC13 E4K	4	E	K	1	none	0	
ANAPC13 E21Q	21	E	Q	1	none	0	
ANAPC13 M55I	55	M	I	2	none	0	
